# Discrimination of three genetically close *Aspergillus* species by using high resolution melting analysis applied to indoor air as case study

**DOI:** 10.1186/s12866-017-0996-4

**Published:** 2017-04-04

**Authors:** Xavier Libert, Ann Packeu, Fabrice Bureau, Nancy H. Roosens, Sigrid C. J. De Keersmaecker

**Affiliations:** 1 Platform Biotechnology and Molecular Biology, Scientific Institute of Public Health (WIV-ISP), J. Wytsmanstraat 14, 1050 Brussels, Belgium; 2 Mycology and Aerobiology, Scientific Institute of Public Health (WIV-ISP), J. Wytsmanstraat 14, 1050 Brussels, Belgium; 3 Cellular and Molecular Immunology, Groupe Interdisciplinaire de Génoprotéomique Appliquée (GIGA), Université de Liège (ULg), Avenue de l’Hôpital, 1 (B34), 4000 Sart-Tilman, Belgium

**Keywords:** *Aspergillus*, High resolution melting analysis, Indoor air, Public health, Molecular method

## Abstract

**Background:**

Indoor air pollution caused by fungal contamination is suspected to have a public health impact. Monitoring of the composition of the indoor airborne fungal contaminants is therefore important. To avoid problems linked to culture-dependent protocols, molecular methods are increasingly being proposed as an alternative. Among these molecular methods, the polymerase chain reaction (PCR) and the real-time PCR are the most frequently used tools for indoor fungal detection. However, even if these tools have demonstrated their appropriate performance, some of them are not able to discriminate between species which are genetically close. A solution to this could be the use of a post-qPCR high resolution melting (HRM) analysis, which would allow the discrimination of these species based on the highly accurate determination of the difference in melting temperature of the obtained amplicon. In this study, we provide a proof-of-concept for this approach, using a dye adapted version of our previously developed qPCR SYBR®Green method to detect *Aspergillus versicolor* in indoor air, an important airborne fungus in terms of occurrence and cause of health problems. Despite the good performance observed for that qPCR method, no discrimination could previously be made between *A. versicolor*, *Aspergillus creber* and *Aspergillus sydowii*.

**Methods:**

In this study, we developed and evaluated an HRM assay for the discrimination between *A. versicolor*, *Aspergillus creber* and *Aspergillus sydowii*.

**Results:**

Using HRM analysis, the discrimination of the 3 *Aspergillus* species could be made. No false positive, nor false negatives were observed during the performance assessment including 20 strains of *Aspergillus*. The limit of detection was determined for each species i.e., 0.5 pg of gDNA for *A. creber* and *A. sydowii*, and 0.1 pg of gDNA for *A. versicolor*. The HRM analysis was also successfully tested on environmental samples.

**Conclusion:**

We reported the development of HRM tools for the discrimination of *A. versicolor*, *A. creber* and *A. sydowii*. However, this study could be considered as a study case demonstrating that HRM based on existing qPCR assays, allows a more accurate identification of indoor air contaminants. This contributes to an improved insight in the diversity of indoor airborne fungi and hence, eventually in the causal link with health problems.

**Electronic supplementary material:**

The online version of this article (doi:10.1186/s12866-017-0996-4) contains supplementary material, which is available to authorized users.

## Background

Today, the contamination of indoor air of buildings by fungi is suggested to be associated with public health problems [1]. However, the causal link between fungal air contamination and respiratory problems is still not well understood. This is partly due to the issues related to the detection and identification of fungal species in indoor air. Indeed classically, the detection and monitoring workflow of indoor fungal contamination are based on the microscopic identification of fungi obtained after a cultivation step [2, 3]. This culture-dependent workflow leads to some bias in the diversity observed due to e.g., species competition on plate, uncultivable species or dead fungi [2–5]. However, these by classical workflow undetected species could affect human health [5]. To avoid this bias, the use of culture-independent, molecular techniques seems to be more advantageous than the classical workflow [4, 6]. That is why PCR [7, 8] and real-time PCR (qPCR) are currently increasingly used for the monitoring of indoor airborne fungi [7, 9–12].

Although qPCR methods are specific and allow the identification up to species level, genetically close species are sometimes difficult to be discriminated using these molecular tools. For example, we previously proposed a qualitative qPCR SYBR®Green method targeting the ITS2 region (*Aversi_ITS* assay) for the detection of *Aspergillus versicolor* [13]*,* an important indoor fungal contaminant [2, 14, 15]. Among the 10 species from the indoor air background that had been included in the specificity test, this tool developed for the specific detection of *A. versicolor* resulted in false positives only for the DNA of 2 genetically close species, i.e., *Aspergillus creber* and *Aspergillus sydowii*, belonging to the same group of *Versicolores*.

As previously elaborated [13], these 3 species are difficult to be discriminated, both morphologically as genetically. The available Taqman assays of the United States Environmental Protection Agency (EPA) for the specific detection of *A. versicolor* and *A. sydowii* respectively, also amplify both species each time [11, 16]. For *A. creber*, no Taqman assays have been developed and/or tested yet. Nevertheless, it has been observed that closely related species could show different antifungal patterns, which is important information to choose the appropriate therapeutic regime [17]. Additionally, Jurjeciv et al. [18] reported that, in a same environmental context, the different species belonging to this *Aspergillus* group (including *A. creber* and *A. versicolor*) produce different concentrations of sterigmatocystin, a precursor of the aflatoxin B1 which is a well-known carcinogenic mycotoxin. *A. sydowii* is known as a non-sterigmatocystin producer [19]. These observations indicate that these 3 genetically closely related species belonging to the *Versicolores* group could have a different effect on health. However, it has not yet been investigated what the difference is concerning the impact of their presence in indoor air on public health. Hereto, a rapid, culture-independent discriminative method is currently lacking. Therefore, this is an interesting case study for the development of a molecular method that can discriminate genetically close species of indoor airborne fungi.

In the present study, we developed a molecular method based on our previously proposed SYBR®Green qPCR method for the detection of *A. versicolor* [13], for the discrimination of *A. versicolor*, *A. sydowii* and *A. creber*. Indeed, the advantage of SYBR®Green includes the possibility to discriminate different amplicons based on their melting temperature (T_m_). However, despite nucleotide variations between the 3 amplicons obtained for respectively *A. versicolor*, *A. sydowii* and *A. creber*, poor discrimination could be made with a classical melting curve analysis [13]. In this context, the technology of high resolution melting (HRM) could offer a good alternative method for the discrimination of species closely related at the genetic level. Unlike the SYBR®Green chemistry, the dye used for HRM analysis is a saturating dye, such as the Evagreen dye. Consequently, all amplicons obtained after the DNA template amplification are saturated by the dye improving the detection of nucleotide variations, in combination with a high resolution qPCR instrument allowing a very detailed analysis of the melting behavior [20]. Based on this particularity, genetically closely related species can be distinguished with HRM analysis as it was shown for *Candida* species and some other invasive fungal species [21–23] including some *Aspergillus* species [24]. The HRM analysis groups together (i.e., clusters) samples with similarities in the shape of the melting curves, which is the outcome of an HRM experiment. By including positive controls for each of the expected species, the discrimination in species-specific clusters can be done.

By taking 3 *Aspergillus* species closely related at the genetic level as a case study, we deliver the proof-of-concept that existing SYBR®Green qPCR methods can be further developed using HRM into more discriminating molecular methods. These offer the possibility to improve the identification of indoor airborne fungi, thereby eventually contributing to establishing the causal link between these contaminants and adverse health effects.

## Methods

### Strains, culturing and DNA isolation

All the species and strains used in this study were previously used to develop the qPCR SYBR®Green *Aversi_ITS* assay [13]. All of these strains were purchased from the BCCM/IHEM collection (Brussels, Belgium) and are listed in Table 1, i.e., *A. creber, A. sydowii*, *A. versicolor* and *P. chrysogenum*. Culturing and the DNA extraction protocols were previously described in Libert et al. [13].

**Table 1:** Species discrimination by HRM analysis

Genus	Species	Reference BCCM/IHEM ^a^	T_m_ mean ± SD (°C) ^b^	Cluster ^c^	Confidence mean ± SD ^d^ (%)
*Aspergillus*	*versicolor*	IHEM 1323	76.45 ± 0.07	1	98.4 ± 0.4
*Aspergillus*	*versicolor*	IHEM 1355	76.30 ± 0.14	1	97.6 ± 2.2
*Aspergillus*	*versicolor*	IHEM 2023	76.40 ± 0.28	1	98.9 ± 0.9
*Aspergillus*	*versicolor*	IHEM 2157	76.35 ± 0.21	1	98.6 ± 0.4
*Aspergillus*	*versicolor*	IHEM 2983	76.30 ± 0.14	1	98.6 ± 1.0
*Aspergillus*	*versicolor*	IHEM 6598	76.40 ± 0.28	1	98.2 ± 0.4
*Aspergillus*	*versicolor*	IHEM 9674	76.55 ± 0.07	1	98.9 ± 0.4
*Aspergillus*	*versicolor*	IHEM 10351	76.30 ± 0.14	1	99.0 ± 0.5
***Aspergillus***	***versicolor***	**IHEM 18884**	76.40 ± 0.28	1	98.6 ± 0.9
*Aspergillus*	*versicolor*	IHEM 19014	76.35 ± 0.21	1	98.8 ± 0.6
*Aspergillus*	*versicolor*	IHEM 19210	76.40 ± 0.01	1	98.5 ± 0.9
*Aspergillus*	*versicolor*	IHEM 19256	76.40 ± 0.28	1	98.8 ± 0.2
*Aspergillus*	*versicolor*	IHEM 22014	76.35 ± 0.21	1	98.3 ± 1.1
*Aspergillus*	*versicolor*	IHEM 22975	76.30 ± 0.14	1	98.6 ± 1.0
*Aspergillus*	*versicolor*	IHEM 24424	76.50 ± 0.42	1	99.0 ± 0.5
*Aspergillus*	*sydowii*	IHEM 895	76.35 ± 0.21	2	99.1 ± 0.4
*Aspergillus*	*sydowii*	IHEM 1360	76.40 ± 0.00	2	99.2 ± 0.3
***Aspergillus***	***sydowii***	**IHEM 20347**	76.50 ± 0.14	2	99.8 ± 0.1
***Aspergillus***	***creber***	**IHEM 2646**	76.40 ± 0.28	3	97.9 ± 1.2
*Aspergillus*	*creber*	IHEM 2916	76.38 ± 0.08	3	98.9 ± 1.3
*Penicillium*	*chrysogenum*	IHEM 20859	/	ND	/
*Penicillium*	*chrysogenum*	IHEM 4151	/	ND	/

The strains in bold are considered as a reference used for the assay development and are fully characterized as respectively *A. creber*, *A. sydowii* and *A. versicolor*. ^a^ Pure strains from the BCCM/IHEM collection. ^b^ Average of the T_m_ ± standard deviation (SD) obtained for each strain analyzed in duplicate during 2 independent runs. ^c^ Cluster 1, 2 and 3 were defined with the Biorad Precision Melt Analysis software 1.2 (Temse, Belgium). ND: not detected. ^d^ Average of the percentage of confidence (± standard deviation SD) from the mean of the cluster, defined with the Biorad Precision Melt Analysis software 1.2 (Temse, Belgium). The percentage of confidence threshold was defined as 95%, below this threshold the result is considered as not acceptable as a true positive

### qPCR and high resolution melting (HRM) conditions

The HRM assays were performed using a CFX96 Touch™ Real-Time PCR Detection System and the CFX manager 3.1 software (Biorad, Temse, Belgium).

The qPCR program was previously described and optimized [13]. The following thermal cycling conditions were used i.e., 1 cycle at 95 °C for 2 min for the complete activation of the hot-start DNA polymerase, 40 cycles at 95 °C for 10 s for the denaturing step, followed by one step at 60 °C for 30 s (annealing and extension), and a final extension at72 °C for 30 s. The PCR amplification was followed by the HRM analysis which is performed in 2 stages, adapted from the instruction manual for the Precision Melt Supermix (Biorad, Temse, Belgium). The first stage was the heteroduplex formation, i.e. 95 °C for 30 s and 60 °C for 1 min. The second step was the high resolution melting (HRM) itself between 63 and 95 °C with an increment of 0.10 °C each 10 s.

As recommended by the manufacturer, the reaction mix (20 μl final volume) contained 10 μl of Precision Melt Supermix with Evagreen dye (Biorad, Temse, Belgium), 1.2 μl of *Aversi_ITS* f and *Aversi_ITS* r (Eurogentec, Liège, Belgium) at 300 nM final concentration each [13], and 2.6 μl of Gibco® DNase, RNase, Protease free pure water (Life Technologies, Gent, Belgium). In each well, an equal amount of 5 μl of each genomic DNA (gDNA) template (1 ng per μl, so 5 ng gDNA in total per well) was added to the reaction mix. During the optimization phase of the HRM assay, 5 ng of gDNA was analyzed in duplicate, each in two independent runs. In each assay a non DNA template control (NTC_water_) composed of Gibco® DNase, RNase, Protease free pure water (Life Technology, Gent, Belgium) and 3 positive controls i.e., *A. creber* IHEM 2646 (5 ng of gDNA), *A. versicolor* IHEM 18884 (5 ng of gDNA) and *A. sydowii* IHEM 20347 (5 ng of gDNA) were added.

### HRM data analysis

The melt-curve data were analyzed with the Biorad Precision Melt Analysis software 1.2 (Biorad, Temse, Belgium).

A sample is defined as positive for a specific species, if an amplicon is obtained, if the observed T_m_ corresponds to the T_m_ defined by Libert et al. [13] for *A. versicolor* (i.e., 76.5 ± 0.18 °C) and if the sample is classified in the same cluster as the cluster defined for its respective positive control. The software also calculates a percent confidence. This value provides a percentage chance that a given well is correctly categorized within the assigned cluster. It is based on the number of standard deviations the sample is from the mean of the cluster. This assumes that the found “cluster means and standard deviations” are accurate descriptions of the real probability distributions of the data [25]. The threshold of the percent confidence was fixed at 95%. Below this limit, the sample was considered as not acceptable as a true positive.

**Fig. 1:**
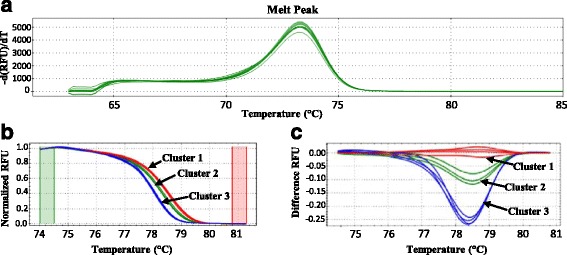
High resolution melting analysis plots. HRM analysis results are illustrated with (**a**) melt peak curves, (**b**) the normalized melt curves and (**c**) the difference plots. Each cluster was defined with the Biorad Precision Melt Analysis software 1.2 (Temse, Belgium). The red curves correspond to *A. versicolor* strains, the green curves to *A. creber* strains and the *blue* curves to *A. sydowii*. gDNA of each strain, i.e., *A. creber* IHEM 2646 (5 ng of gDNA), *A. versicolor* IHEM 18884 (5 ng of gDNA) and *A. sydowii* IHEM 20347, was tested in duplicate in 2 independent runs. RFU: relative fluorescence units

The clustering of each sample can be visualized by the software with different charts, e.g. the melt peak curve, the normalized melt curve chart and the difference curve chart. The melt peak curve (Fig. 1a) shows the derivative of the fluorescence versus temperature, indicating the T_m_. The normalized melt curve (Fig. 1b) shows a normalized view of the melt curve of each sample (Pre-melt (initial) and post-melt (final) fluorescence signals of all samples are normalized to relative values of 100% and 0%, differences in background fluorescence between curves are eliminated) and plots their relative fluorescence unit (RFU) against the temperature. The difference curve (Fig. 1c) magnifies curve differences by subtracting each curve from the most abundant type or from a user-defined reference. By setting a baseline, small differences between the RFU obtained for each cluster become visible.

### Sensitivity test: limit of detection

To evaluate the sensitivity of the use of the *Aversi_ITS* f/r primers for the HRM application, a serial dilution of gDNA of *A. creber* BCCM/IHEM 2646, *A. sydowii* BCCM/IHEM 20347 and *A. versicolor* IHEM 18884, defined as a reference by Libert et al. [13] for the performance assessment of the *Aversi_ITS* assay, was made and the limit of detection (LOD) was determined. A serial of 10 dilutions from 1000 to 0.01 pg (i.e., 1000, 500, 50, 10, 5, 1, 0.5, 0.1, 0.05, 0.01 pg) was analyzed in duplicate with 6 independent runs. To comply with the Precision Melt Supermix requirements on the minimum amount of DNA to be present in the well, all the dilutions were made in 10 ng deoxyribonucleic acid sodium salt from salmon testes i.e., salmon sperm DNA (Sigma-Aldrich, Diegem, Belgium). In each assay, a negative control (NTC_salmon sperm_), i.e., 10 ng of salmon sperm DNA (Sigma-Aldrich, Diegem, Belgium) and NTC (composed of Gibco® DNase, RNase, Protease free pure water) were added. The LOD was determined as the lowest amount of gDNA where for at least 11 out of the 12 repetitions the amplicon (with the correct T_m_) and the correct cluster (with confidence >95%) were obtained.

### Symmetric and asymmetric DNA concentration test

In order to evaluate the discriminative power of the HRM assay, 12 mixes of gDNA of *A. creber, A. sydowii* and *A. versicolor* were analyzed in duplicate. The mixes’ composition is presented in the first part of Table 3. Briefly, first 5 ng of gDNA from a mix (A) containing an equal amount of gDNA from each targeted species (i.e., 5 ng of each species for 5 μl of total volume of mix) that were mixed together (Table 3) was analyzed with HRM. To assess the capacity of the HRM assay to detect and discriminate the 3 targets in an imbalanced mix of gDNA, as it could occur in environmental samples, a second (B to G) set of mixes was analyzed (Table 3). In mixes B to D, 2 species were introduced with an equal amount of gDNA (i.e., 5 ng both for 5 μl of total volume of mix) and a third one at the LOD; in mixes E to G, one species dominated the others (i.e., 25 ng for 5 μl of total volume of mix), while the 2 others were added at the same amount of gDNA (i.e., 5 ng both for 5 μl of total volume of mix). Each HRM analysis was performed in duplicate with 5 μl of gDNA mix. The strains used for the mixes were the BCCM/IHEM 2646 for *A. creber*, the BCCM/IHEM 895 for *A. sydowii* and the BCCM/IHEM 10351 for *A. versicolor*.

In each assay, an NTC (no DNA) and 3 positive controls were added to the assay i.e., *A. creber* IHEM 2646 (5 ng of gDNA), *A. versicolor* IHEM 18884 (5 ng of gDNA) and *A. sydowii* IHEM 20347 (5 ng of gDNA). At the time of these experiments, the strain BCCM/IHEM 2646 was the only confirmed strain of *A. creber* available in the collection. During the redaction of this manuscript, a new *A. creber* strain (BCCM/IHEM 2916) was added to the BCCM/IHEM collection. To verify whether a correct discrimination could be done for this new strain, a test was performed following the conditions described in the sections qPCR and high resolution melt conditions and HRM data analysis (Table 1). The results matched with those previously obtained with the strain BCCM/IHEM 2646. Therefore it was decided that the strain *A. creber* BCCM/IHEM 2646 could be used as a representative for *A. creber* in the symmetric and asymmetric concentration tests.

### Specificity assessment

In order to verify that the discrimination of the 3 targeted species (*A. creber, A. sydowii*, *A. versicolor*) can be done in the presence of a non-targeted species (*P. chrysogenum*), 3 additional mixes (H to J, Table 3) were tested, where *P. chrysogenum* replaced one of the 3 *Aspergillus* species. The pure strain *P. chrysogenum* BCCM/IHEM 20849 was selected as a negative control. The mixes H, I and J have equal composition containing 5 ng per *Aspergillus* species and 25 ng for *P. chrysogenum* (Table 3). The mix K was introduced as a negative control containing only 5 ng of *P. chrysogenum* gDNA extracted from the strain BCCM/IHEM 20849 (Table 3). In each assay, an NTC_water_ and 3 positive controls were added i.e., *A. creber* IHEM 2646 (5 ng of gDNA), *A. versicolor* IHEM 18884 (5 ng of gDNA) and *A. sydowii* IHEM 20347 (5 ng of gDNA). The strains used for the mixes were the BCCM/IHEM 2646 for *A. creber*, the BCCM/IHEM 895 for *A. sydowii* and the BCCM/IHEM 10351 for *A. versicolor*.

### Proof-of-concept using environmental air samples

To assess the performance of the *Aversi_ITS* assay to discriminate the 3 targeted *Aspergillus* spp. using the HRM technology, 4 environmental samples previously collected in a single contaminated house and previously analyzed with the *Aversi_ITS* assay and classical identification methods [13], were used. The sampling protocol, the classical method of identification (i.e., counting and microscopic identification) and the gDNA extraction have been previously described [13]. The HRM analysis was performed in duplicate in 4 independent repeats, as described above. In addition to using the DNA extracted from the environmental samples as such, in a second round of experiments, in order to avoid any issues with the HRM supermix, 10 ng of salmon sperm DNA was added to all DNA mixtures prior to HRM analysis. As mentioned above, each HRM analysis included a positive control for each of the species to be discriminated (25 ng of gDNA).

To be sure that no inhibition occurs during the HRM analysis and in order to verify that all the 3 species can be detected and discriminated with the HRM analysis, two sets of gDNA from pure cultures were spiked into one environmental sample where *A. versicolor* was not detected by classical nor qPCR methods (i.e., sample 3). A first set contained 25 ng gDNA extracted from pure culture (*A. creber* 2646 IHEM/BCCM, *A. sydowii* 895 and *A. versicolor* 10,351) spiked into the DNA extracted from the environmental sample 3. In addition, spikes were made with an amount of DNA for each of the targeted species at the LOD in order to verify whether a very small amount could be detected in this environmental sample. Similarly to what was done with the ‘pure’ environmental samples, a second set of sample was made with an addition of salmon sperm DNA (10 ng) into the spiked samples in order to increase the DNA amount available for the supermix (cfr. The sensitivity test). As mentioned above, in each assay, a NTC_water_, a NTC_salmon sperm_ and 3 positive controls were added i.e., *A. creber* IHEM 2646 (25 ng of gDNA), *A. versicolor* IHEM 18884 (25 ng of gDNA) and *A. sydowii* IHEM 20347 (25 ng of gDNA).

## Results

### HRM assay development

The HRM analysis reported in this study is based on a previously published qPCR SYBR®Green assay [13], called *Aversi_ITS*. In that study [13], during the performance assessment, the specificity was tested for 10 species frequently found in indoor air i.e., *Alternaria alternata*, *A. creber*, *Aspergillus fumigatus*, *A. sydowii*, *A. versicolor*, *Cladopsorium cladosporioides*, *Cladosporium herbarum*, *Penicillium chrysogenum*, *Stachybotrys chartarum* and *Ulocladium botrytis*. This test showed to be specific for *A. versicolor*, i.e. no amplification for the non-targeted species, with the exception of *A. creber* and *A. sydowii* which yielded an amplicon with the same T_m_ than the one observed for *A. versicolor* (i.e., 76.5 °C).

Therefore, the species discrimination using the *Aversi_ITS* assay was optimized using the HRM approach as elaborated in Materials and Methods for the species for which amplification was obtained with the *Aversi_ITS* assay. Hereto, firstly, the HRM assays were performed in duplicate using gDNA extracted from pure strains acting as reference strains in Libert et al. [13] i.e., the BCCM/IHEM strain 18,884 for *A. versicolor*, the BCCM/IHEM strain 20,347 for *A. sydowii* and the BCCM/IHEM strain 2646 for *A. creber*. The 3 reference strains showed an expected melting temperature (T_m_) ranging between 76.40 ± 0.28 and 76.50 ± 0.14 and could be classified each in a different cluster (Fig. 1a, b, c ; Table 1). Cluster 1 grouped together all the replicates of the *A. versicolor* BCCM/IHEM 18884 with a confidence of 98.6 ± 0.9. Cluster 2 was defined by all the replicates from the *A. sydowii* BCCM/IHEM 20347 strain with a confidence of 99.8 ± 0.1. Finally, all the replicates from the *A. creber* BCCM/IHEM strain 2646 were classified in cluster 3 with a confidence of 97.9 ± 1.2.

Subsequently, all other strains available in the BCCM/IHEM collection and belonging to the species *A. creber, A. versicolor* or *A. sydowii* were tested. The T_m_ of the obtained amplicon was determined to verify the PCR amplification step. As previously reported [13], gDNA of all species tested resulted in an amplicon with a T_m_ around 76.5 ± 0.18 °C (Table 1). No amplification was observed in any of the NTCs added to the assays. All the strains of *A. versicolor* were grouped in cluster 1 defined by the reference strain BCCM/IHEM 18884 with a confidence defined between 97.6 ± 2.2% and 99.0 ± 0.5%. Those of *A. sydowii* were grouped in cluster 2 defined by the reference strain BCCM/IHEM 20347 with a confidence ranging between 99.1 ± 0.4% and 99.2 ± 0.3% and those of *A. creber* in cluster 3 defined by the reference strain BCCM/IHEM 2646 with 98.9 ± 1.3% of confidence. In Additional file 1, the normalized melt curves and difference curves for each of the tested strain have been depicted to illustrate the inter- and intra-species variabilities. These results are in line with what was expected based on the alignment of the amplified region in each of the strain tested (Additional file 2).

*P. chrysogenum* was tested as negative control because of its phylogenetic proximity with the *Aspergillus* genus [26] and its importance in indoor air contamination [10, 27]. Two different strains were included, but they did not yield an amplicon as expected based on the selectivity previously determined for the *Aversi_ITS* assay [13].

### Sensitivity test: limit of detection

To define the lowest amount of gDNA that can be discriminated with the HRM analysis, a serial dilution, from 1000 to 0.01 pg in 10 steps repeated in duplicate in 6 independent runs was tested (Table 2). Because the HRM supermix cannot be used with an amount of input DNA lower than 0.1 ng, all the dilutions were made in salmon sperm DNA. No amplification was observed in the negative controls i.e., NTC_water_ and NTC_salmon sperm_ (data not shown). For *A. versicolor*, the LOD of the *Aversi_ITS* qPCR assay was previously defined at 1 copy of gDNA [13] which corresponds to 0.05 pg of gDNA. In the HRM assay, *A. versicolor* was amplified until 0.1 pg of gDNA with 12/12 positive detection events and until 0.05 pg of gDNA with 10/12 positive detections, i.e. correct amplicon and cluster and confidence >95%. Below this limit of 0.05 pg, no amplification was observed for *A. versicolor* in the tested concentrations. *A. creber* and *A. sydowii* were detected with minimum 11/12 positive detection events until 0.5 pg of gDNA (Table 2). As shown in Table 2, the discrimination in the 3 different clusters could be made with HRM until 0.5 pg of gDNA, under this limit no discrimination could be made between *A. creber* and *A. sydowii*. Based on these observations, the LOD for the HRM assay was defined at 0.5 pg of gDNA for *A. creber* and *A. sydowii* and at 0.1 pg for *A. versicolor* (Table 2).

**Table 2:** Limit of detection of HRM assay

Parameter	Genus	Species ^a^	Cluster ^b^	gDNA amount (pg)									
				1000	500	50	10	5	1	0.5	0.1	0.05	0.01
Detection ^c^	*Aspergillus*	*creber*	3	12/12	12/12	12/12	12/12	12/12	12/12	**11/12**	6/12	0/12	0/12
	*Aspergillus*	*sydowii*	2	12/12	12/12	12/12	12/12	12/12	12/12	**11/12**	9/12	0/12	0/12
	*Aspergillus*	*versicolor*	1	12/12	12/12	12/12	12/12	12/12	12/12	12/12	**12/12**	10/12	0/12
Confidence (%) ^d^	*Aspergillus*	*creber*	3	97.98 ± 1.28	96.66 ± 1.54	99.03 ± 0.59	99.30 ± 0.29	99.70 ± 0.02	97.99 ± 0.53	96.65 ± 0.36	97.99 ± 0.53.	/	/
	*Aspergillus*	*sydowii*	2	98.72 ± 0.39	99.25 ± 0.26	99.28 ± 1.66	99.67 ± 1.12	98.840 ± 2.34	96.83 ± 3.11	95.63 ± 1.63	95.83 ± 3.11	/	/
	*Aspergillus*	*versicolor*	1	98.99 ± 0.26	99.23 ± 0.35	99.49 ± 2.01	98.93 ± 0.60	99.76 ± 096	98.52 ± 2.32	97.49 ± 0.11	97.60 ± 1.06	96.37 ± 1.540	/

^a^gDNA extracted from pure strains from the BCCM/IHEM collection. BCCM/IHEM strains used as reference: *A. creber* IHEM 2646, *A. sydowii* IHEM 26347, *A. versicolor* IHEM 18884

^b^Cluster defined with the Biorad Precision Melt Analysis software 1.2 (Temse, Belgium)

^c^Detection: Number of positive signals i.e., correct cluster and confidence >95%, observed for each dilution, the LOD for each species is indicated in bold

^d^Confidence (%) is the average and standard deviation (±SD)

### Symmetric and asymmetric DNA concentration test

The capacity to detect and to discriminate the 3 *Aspergillus* species when they are mixed, was tested with two different types of mix composition i.e., a symmetric mix including an equal amount of gDNA from each targeted species (*A. creber*, *A. sydowii* and *A. versicolor*) and a asymmetric mix composed of a different amount of gDNA from the 3 *Aspergillus* species, including one species present at LOD, and mixes that contained the negative control *P. chrysogenum*.

The HRM analysis of the symmetric mix A (i.e., a mix of 5 ng of gDNA of each *Aspergillus* species) yielded 3 different peaks in the Melt Peak Chart, reflecting the presence of the 3 targeted *Aspergillus* species (Table 3)*.* The melting profiles were clustered in another cluster than the ones corresponding to the positive controls where only one target species was present, each time with a confidence % above 95% (Table 3).

**Table 3:** Symmetric, asymmetric and specificity assessment of different mixes

Parameter	Species ^a^	Mix										
		A	B	C	D	E	F	G	H	I	J	K
DNA amount (ng) ^b,c^	*A. creber*	5	LOD^c^	5	5	25	5	5	0	5	5	0
	*A. sydowii*	5	5	LOD	5	5	25	5	5	0	5	0
	*A. versicolor*	5	5	5	LOD	5	5	25	5	5	0	0
	*P. chrysogenum*	0	0	0	0	0	0	0	25	25	25	5
Cluster ^d^		4	4	4	4	4	4	4	5	6	7	ND
Peaks in Melt Peak Chart observed ^e^		72.40 ± 0.00	72.40 ± 0.00	72.40 ± 0.00	72.40 ± 0.00	72.40 ± 0.00	72.50 ± 0.14	72.50 ± 0.14	78.40 ± 0.00	77.20 ± 0.28	78.10 ± 0.14	ND
		80.60 ± 0.00	80.60 ± 0.00	80.60 ± 0.00	80.60 ± 0.00	80.60 ± 0.00	80.60 ± 0.00	80.60 ± 0.00	81.60 ± 0.00	85.00 ± 0.00	82.40 ± 1.41	
		85.40 ± 0.00	85.40 ± 0.00	85.60 ± 0.00	85.60 ± 0.00	85.50 ± 0.14	85.40 ± 0.00	85.40 ± 0.00				
Confidence (%) ^f^		99.85 ± 0.07	99.75 ± 0.07	99.55 ± 0.35	96.65 ± 2.33	99.85 ± 0.07	99.85 ± 0.07	99.25 ± 1.06	99.30 ± 0.00	99.60 ± 0.00	99.10 ± 0.56	ND

^a^gDNA extracted from pure strains from the BCCM/IHEM collection

^b^Each assay contained 4 pure strains i.e., BCCM/IHEM 2646 for *A. creber*, BCCM/IHEM 895 for *A. sydowii*, BCCM/IHEM 10351 for *A. versicolor*, and BCCM/IHEM 4151 for *P. chrysogenum*. The former 3 were used as positive control and resulted in cluster 1 (*A. versicolor*), cluster 2 (*A. sydowii*) and cluster 3 (*A. creber*). For *P. chrysogenum*, used as negative control, no amplification and hence no cluster was obtained. Each HRM reaction was done with 5 μl of mix

^c^LOD defined as the limit of detection defined for each species i.e., 0.1 pg for *A. versicolor* and 0.5 pg for *A. creber* and *A. sydowii*

^d^Detection parameters i.e., clusters defined with the Biorad Precision Melt Analysis software 1.2 (Temse, Belgium). ND: no amplicon obtained, no cluster detected

^e^Peak observed in the Melt Peak chart given by the Biorad Precision Melt Analysis software 1.2 (Temse, Belgium) – this does not correspond to the Tm of each separate amplicon of the different species

^f^Confidence (%) is the average of each percentage of confidence defined with the Biorad Precision Melt Analysis software 1.2 (Temse, Belgium) obtained for each repetition and standard deviation (±SD)

In the 3 first asymmetric mixes (i.e., mixes B, C and D, Table 3), the amount of gDNA from one of the 3 *Aspergillus* species was taken at the LOD previously defined i.e., 0.1 pg for *A. versicolor* and 0.5 pg for the 2 others. The results obtained were similar (i.e., 3 peaks, correct cluster, confidence % in the same range) to those observed with the symmetric mix, even when the amount of gDNA was at the LOD (Table 3). In the mixes E, F and G, one of the 3 species was added in a higher amount than that of the other 2. Once again, the 3 peaks and the cluster corresponding to the mixed species were found with high confidence with all mix configurations (Table 3).

### Specificity assessment

Although the *Aversi_ITS* assay was previously shown to be specific for *A. versicolor*, as evaluated for the 10 most frequent species from the indoor air background, the discrimination between *A. versicolor*, *A. creber* and *A. sydowii* was not observed in classical qPCR [13]. To evaluate that the discrimination of these 3 *Aspergillus* species by the HRM method is not influenced by the presence of non-targeted species, even at a dominant concentration, 3 others mixes were made and analyzed with HRM.

In mixes H, I and J, one of the targeted species was removed from the mix (Table 3), and replaced by 25 ng of *P. chrysogenum* gDNA and analyzed with the *Aversi_ITS* HRM method. For each of the mixes, we obtained 2 peaks in the Melt Peak Chart (Table 3). Each of the mixes was assigned another cluster than the one obtained for the positive controls or the mixes with 3 species, each time with a confidence above 95% (Table 3). For mix K, where *P. chrysogenum* was the only species present, no amplification was obtained, and hence no cluster was assigned (Table 3). This was expected based on the results of Libert et al. [13].

### Proof-of-concept

In order to verify whether this HRM analysis could be used on real-life samples, 4 environmental air samples, previously analyzed by classical methods (i.e., plate counting and microscopic identification) and by qPCR for the detection of *A. versicolor* [13], were re-analyzed with the *Aversi_ITS* HRM assay.

As summarized Table 4, the *Aversi_ITS* HRM analysis defined 3 different clusters for the 3 positive controls with or without the addition of salmon sperm DNA. The 2 NTCs (NTC _water_ and NTC _salmon sperm_) did not yield an amplicon.

**Table 4:** Environmental test

Sample	Sample type	Species ^a^	Amount of DNA/HRM analysis (ng) ^b^	Salmon sperm ^c^	Cluster ^d^	Percentage of confidence (%) ^d^	Number of positive detections ^e^
Control							
NTC _water_	water only	water only	0	No	N/A	/	
NTC_salmon sperm_	Salmon sperm only	Salmon sperm only	10	Yes	N/A	/	
Positive control	Pure culture	*A. creber*	25	No	3	95.74 ± 7.07	4/4
Positive control	Pure culture	*A. creber*	25	Yes	3	97.32 ± 3.90	4/4
Positive control	Pure culture	*A. sydowii*	25	No	2	97.91 ± 3.26	4/4
Positive control	Pure culture	*A. sydowii*	25	Yes	2	98.89 ± 0.97	4/4
Positive control	Pure culture	*A. versicolor*	25	No	1	99.16 ± 0.49	4/4
Positive control	Pure culture	*A. versicolor*	25	Yes	1	97.99 ± 2.38	4/4
Environmental sample							
Sample 1	Indoor air sample	*A. versicolor*	19.8	No	1	99.15 ± 0.08	6/8
				Yes	1	99.76 ± 0.01	7/8
		*P. chrysogenum*					
Sample 2	Indoor air sample	*A. versicolor*	21.7	No	1	98.10 ± 0.88	5/8 ^$^
				Yes	1	98.93 ± 0.45	7/8
		*A. glaucus*					
		*P. chrysogenum*					
		*yeast* (undetermined)					
Sample 3	Indoor air sample	*P. chrysogenum*	53.3	No	N/A		
				Yes	N/A		
Sample 4	Indoor air sample	*A. versicolor*	50	No	1	98.81 ± 1.90	5/8^$^
				Yes	1	98.58 ± 0.55	8/8
		*infertile mycelium*					
		*P. chrysogenum*					

^$^At least one repetition is considered as negative due to a confidence below the 95 % threshold

^a^Determined with classical methods i.e. plate culture and microscopic analysis (determination and counting) in Libert et al. [13]

^b^Amount of DNA extracted from air samples of 1.5 m^3^; DNA amount determined with a Nanodrop® 2000; 5 μl of extracted DNA (5 ng/μl) were used in a 20 μl -HRM analysis

^c^Salmon sperm (salmon sperm) DNA carrier added (10 ng)

^d^Cluster and % of confidence ± standard deviation (SD) defined with the Biorad Precision Melt Analysis software 1.2 (Temse, Belgium)

^e^A sample is defined as positive for a specific species, if an amplicon is obtained, if the observed T_m_ corresponds to the Tm defined by Libert et al. [13] for *A. versicolor* (i.e., 76.5 ± 0.18 °C) and if the sample is classified in the same cluster as the cluster defined for its respective positive control with a confidence >95%

Three environmental samples (i.e., samples 1, 2 and 4), with or without the addition of salmon sperm DNA, gave a positive signal for *A. versicolor* and were all classified in only one cluster corresponding to the one of the *A. versicolor* positive control. The number of positive detections (i.e., amplification and classification in the correct cluster and confidence above 95%) of the 8 repetitions was between 5/8 and 8/8 with a confidence ranging between 98.58 ± 0.55 and 99.76 ± 0.01 (Table 4). The lowest number of positive detections and the lowest confidence were obtained for a sample without the addition of salmon sperm DNA. These results were in accordance with those previously obtained by classical methods and qPCR where *A. versicolor* was found on plate and detected by qPCR [13]. No amplification and consequently no cluster, was observed for the sample 3. This result was also obtained with the classical analysis [13], where no *A. versicolor* was detected on plate (Table 4).

Based on the above described results, it could be concluded that the 3 environmental samples contained the *A. versicolor* species, and not one of the 2 species closely related at the genetic level belonging to the same *Versicolores* group. However, in order to verify that if one of the other 2 species would have been present, it would have been possible to be detected in the environmental sample, a spike test with each of the targeted species was performed as described in Methods. At the highest gDNA concentration, with or without salmon sperm DNA, all the species were detected and classified in the correct cluster defined by the corresponding positive control (Additional file 3). The same observations were made for all spikes at LOD with a confidence between 97.70 ± 1.56% and 99.08 ± 1.14% and a positive rate between 5 and 8 for the 8 repetitions depending on the addition of salmon sperm DNA (Additional file 3: Table).

## Discussion

Currently, an increasing amount of studies are focused on fungal indoor contamination and its impact on public health [9, 10, 15, 28–32]. To rapidly detect and identify the fungal contaminants, qPCR holds a great potential in comparison to the classical methods based on plate counting and microscopy. Indeed, this molecular technique is rapid, sensitive, easy to use and culture independent [4]. However, some issues could occur especially for the discrimination of species closely related at the genetic level. This problem was highlighted by Libert et al. [13] during the development of a SYBR®Green qPCR tool for the detection of *A. versicolor* (*Aversi_ITS*), an important contaminant of indoor environment. Even if this *Aversi_ITS* assay is fast, efficient, sensitive and specific for *A. versicolor* as evaluated for the 10 most frequently occurring fungal species in indoor air, no discrimination between *A. versicolor, A. creber* and *A. sydowii* could be previously made. Indeed the insufficient variation of nucleotides inside their respective amplicons yielded a too similar T_m_ value impeding discrimination by qPCR [13]. Nevertheless, the accurate identification of fungal contaminants is important to eventually determine the causal link between indoor airborne fungal pollution and respiratory health problems.

In this context, to improve the specific detection of *A. versicolor* in indoor air and to discriminate it from the other targets *A. creber* and *A. sydowii*, a post-qPCR HRM was optimized on the basis of the *Aversi_ITS* assay [13]. The HRM analysis developed in this study showed the possibility to improve the *Aversi_ITS* qPCR assay by the discrimination of *A. versicolor* from *A. creber* and *A. sydowii*. All the strains for the 3 species that were available in the BCCM/IHEM collection were not only detected but also classified with a high confidence in 3 different clusters (Table 1, Fig. 1a, b, c), demonstrating the inclusivity and discriminative power of the assay.

The absence of amplification when using gDNA of the 2 *P. chrysogenum* negative controls (Table 1) confirms the exclusivity of the primers of the *Aversi_ITS* assay. These results confirmed those previously shown by Libert et al. [13], during an exclusivity test on 10 species selected as the most detected fungal species in indoor air where no amplification was obtained for the non-targeted species.

During this study, a total of 20 strains of *Aspergillus* were tested, including all strains available for each species in the BCCM/IHEM collection i.e., 2 strains for *A. creber*, 3 for *A. sydowii*, and 15 for *A. versicolor*. In our previous study on the *Aversi_ITS* development [13], we made a sequence alignment with all the sequences of *A. creber*, *A. sydowii* and *A. versicolor* available in the NCBI database at the date of analysis, which corresponds to the alignment with the sequences of all *A. creber*, *A. sydowii* and *A. versicolor* strains used during the development of the HRM assay (Additional file 2). In this study, because these *Aspergillus* species are genetically grouped in the *Versicolores* group, primer exclusivity was also verified using an alignment of primers sequences and all the ITS sequences available in the NCBI database for all the species from this group, i.e. *Aspergillus amoenus, Aspergillus austroafricanus, Aspergillus cvjetkovicii, Aspergillus fructus, Aspergillus jensenii, Aspergillus protuberus, Aspergillus puulaauensis, Aspergillus subversicolor, Aspergillus tabacinus, Aspergillus tennesseensis* and *Aspergillus venenatus*. As these showed several nucleotide variations, i.e. more than 3 as is the case for *A. versicolor*, *A. creber* and *A. sydowii*, inside the amplicon (primers annealing site included) defined by the primers sequences, these species should be able to be discriminated from *A. creber*, *A. sydowii* and *A. versicolor* (based on the T_m_) (Additional files 4 and 5).

The sensitivity for the HRM assay was also tested by defining the LOD for discrimination for each species. For *A. versicolor*, the LOD was previously determined for the *Aversi_ITS* qPCR assay at 1 or 2 copies of gDNA [13], corresponding to 0.05 pg of gDNA of *A. versicolor*. The LOD observed for *A. versicolor* in the HRM assay was determined at 0.1 pg of gDNA. This difference is due to the confidence threshold applied in this study (i.e., 95%). The dissimilarity observed between the LOD for the qPCR and HRM could be explained by the fact that an HRM analysis needs a higher amount of DNA templates to discriminate with high confidence. According to the user guide for the HRM analysis [25], the threshold for an HRM discrimination is observed around 30 C_q_ (corresponding to 0.5 pg of DNA of *A. versicolor*). Above this C_q_ limit, the results are too variable. As no LOD was defined in the previous study for *A. creber* and *A. sydowii*, a sensitivity test was performed for the HRM assay and the LOD for these two species was defined at 0.5 pg of gDNA (Table 2). Thus, as observed by Libert et al. [13] with the SYBR®Green chemistry, the *Aversi_ITS* primers are more efficient for the amplification of gDNA of *A. versicolor* than for the two other species. This difference of sensitivity could impact the level of detection in real-life samples where some species could be present in very low concentration. However, in addition to the sensitivity assessment, HRM assays were performed on gDNA mixes from pure cultures of *A. creber, A. sydowii* and *A. versicolor*, present at different amounts, including at the LOD. The results obtained during these tests demonstrated that the HRM technology can be used to detect a mix of species, even when the gDNA mix is not equilibrated (Table 3). By including the appropriate positive controls (single target and different mixes of 2 and 3 pure strains) the species can be discriminated. However, it has to be mentioned that in real-life samples, the chances to find a mix of the 3 species, i.e. *A. versicolor*, *A. creber* and *A. sydowii* are rather small.

Sensitivity and discrimination were also observed for the environmental, i.e. indoor air samples containing gDNA from these 3 species. One of those samples, which was demonstrated to be negative for *A. versicolor* based on classical methods and qPCR [13], was used to spike the 3 targeted species for HRM analysis. No inhibition from the environmental sample matrix on the detection and discrimination of the 3 species was detected. The discrimination between the 3 species could be made, even if one of them was present at the LOD or if a non-targeted species was present (e.g., *P. chrysogenum)* (Table 4 and Additional file 3).

In the other non-spiked indoor air samples, *A. versicolor* was detected, in accordance with the previous results obtained with classical methods based on culture and microscopic determination (Table 4). In each of these positive samples, *A. versicolor* was present with other common indoor air species (i.e., *A. glaucus* and *P. chrysogenum*) or with undetermined strains. This however did not affect the HRM-based detection, with a detection and discrimination of *A. versicolor* in each of the samples where the SYBR®Green *Aversi_ITS* qPCR method previously detected *A. versicolor* [13]. However, based on the classical detection methods [13], it was observed that the level of contamination by *A. versicolor* in sample 2 was close to the LOD of the qPCR (i.e., 0.05 pg of gDNA). This could explain why the level of positive repetitions in the HRM analysis varied between 62.5% (5/8, without salmon sperm DNA added) and 100% (8/8, with salmon sperm DNA added) (Table 4). This might also indicate that adding salmon sperm DNA to the HRM reactions improves the performance of this assay, as a similar trend was observed for the other environmental samples. However, no statistical evidence could be obtained for this observation due to the low amount of total extracted DNA available per environmental sample for the analysis which limited the number of repetition which could be made.

In comparison to other powerful discriminatory methods such as high-throughput sequencing, the advantage of the HRM analysis is that is a faster (only 1 step, in comparison to multiple steps for sequencing) and more cost-effective method to highly accurately screen a large amount of samples for the presence of specific targets. In our study, HRM allowed to discriminate 3 closely related *Aspergillus* species, thereby offering a tool to investigate in future studies the specific impact of each species on health issues, which was until now not possible with the currently used methods. The HRM method can be easily implemented in a public health laboratory, with an instrument that is not that expensive as and more user-friendly than a sequencer, and it does not require bioinformatics expertise which in contrast is needed to analyze high-throughput sequencing data. Additionally, sequencing errors can have an impact on the interpretation of high-throughput sequencing-based identification methods, especially when the discrimination is based on a single nucleotide difference. So for our application, the HRM analysis is more advantageous than sequencing. For other applications, such as the determination of the diversity in a sample, high-throughput sequencing might be more suited.

## Conclusions

Conclusively, our study, by taking 3 *Aspergillus* genetically closely related species as a case study, demonstrated that HRM analysis, based on existing qPCR methods, could be used to more accurately detect and identify indoor fungal contamination. HRM analysis offers the advantage to easily discriminate genetically close species which are difficult to be distinguished. This increase in accuracy will improve data on indoor air fungal contamination. This is especially important for currently difficult to be discriminated species, but that could however diverge in terms of toxicity, allergenicity or pathogenicity. In this study, the proof of concept was delivered for the 3 species from the *Versicolores* group based on a SYBR®Green *Aversi_ITS* qPCR method previously developed for *A. versicolor* [13]. In the future, other existing SYBR®Green qPCR assays could be adapted for HRM by using a saturating dye for other species, to improve the discrimination of genetically close species. Eventually, the use of HRM in routine analysis performed in the framework of monitoring activities will contribute to the insight in the causal link between indoor fungal contamination and public health.

## Additional files

Additional file 1: Normalized melt curves and difference curves obtained for each *Aspergillus* strain used during the development of the HRM assay (XLSX 105 kb)

Additional file 2: Alignment of the 20 *Aspergillus* strains used for the HRM development (XLSX 3516 kb)

Additional file 3: Spike test results (XLSX 12 kb)

Additional file 4: Alignment of the 9 *Versicolores* species and the *Aversi_ITS* primers (XLSX 3495 kb)

Additional file 5: Melting temperature obtained for the species from the *Versicolores* group (XLSX 11 kb)

## Abbreviations

EPA: Environmental Protection Agency
gDNA: genomic DNA
HRM: high resolution melting
ITS: internal transcribed spacer
LOD: limit of detection
NTC: No Template Control
PCR: polymerase chain reaction
qPCR: real-time PCR
RFU: relative fluorescence units
T_m_: melting temperature

## References

[1] World Health Organization (2009). WHO guidelines for indoor air quality: dampness and mould.

[2] Beguin H, Nolard N (1994). Mould biodiversity in homes. I. Air and surface analysis of 130 dwellings. Aerobiologia.

[3] Nolard N, Chasseur C, Marlier M, Lognay G. Validation des méthodes microbiologiques et chimiques de contrôle des lieux de travail. 2004. http://www.belspo.be/belspo/organisation/publ/pub_ostc/PS/rPS19_fr.pdf. Accessed 20 Mar 2016.

[4] Pitkaranta M, Meklin T, Hyvarinen A, Nevalainen A, Paulin L, Auvinen P (2011). Molecular profiling of fungal communities in moisture damaged buildings before and after remediation-a comparison of culture-dependent and culture-independent methods. BMC Microbiol.

[5] HUD. Controlling and preventing household mould and moisture problems, lessons learned and strategies for disseminating best practices. Report to Congress. April 1, 2005. US HUD. 2006. Healthy Homes Issues: Residential Assessment.

[6] Vesper S (2011). Traditional mould analysis compared to a DNA-based method of mould analysis. Crit Rev Microbiol.

[7] Martin K, Rygiewicz P. Fungal-specific PCR primers developed for analysis of the ITS region of environmental DNA extracts. BMC Microbiol. 2005;5:28. 10.1186/1471-2180-5-28 PMC1156903 15904497

[8] Zhou G, Whong W-Z, Ong T, Chen B (2000). Development of a fungus-specific PCR assay for detection low-level fungi in an indoor environment. Mol Cell Probes.

[9] Bellanger AP, Reboux G, Roussel S, Grenouillet F, Didier-Scherer E, Dalphin JC (2009). Indoor fungal contamination of moisture-damaged and allergic patient housing analysed using real-time PCR. Lett Appl Microbiol.

[10] de Ana SG, Torres-Rodriguez JM, Ramirez EA, Garcia SM, Belmonte-Soler J (2006). Seasonal distribution of *Alternaria*, *Aspergillus*, *Cladosporium* and *Penicillium* species isolated in homes of fungal allergic patients. J Investig Allergol Clin Immunol.

[11] Haugland RA, Varma M, Wymer LJ, Vesper SJ (2004). Quantitative PCR analysis of selected *Aspergillus*, *Penicillium* and *Paecilomyces* species. Syst Appl Microbiol.

[12] Morrison J, Yang C, Lin K-T, Haugland R, Neely A, Vesper S (2004). Monitoring *Aspergillus* species by quantitative PCR during construction of a multi-storey hospital building. J Hosp Infect.

[13] Libert X, Bladt S, Chasseur C, Bureau F, Roosens NH, De Keersmaecker SCJ (2015). Development and performance assessment of a qualitative SYBR®green real-time PCR assay for the detection of *Aspergillus versicolor* in indoor air. Appl Microbiol Biotechnol.

[14] Andersen B, Frisvad J, Rasmussen IS, Larsen L (2011). Association between fungal species and water-damaged building materials. Appl Environ Microbiol.

[15] Packeu A, Chasseur C, Bladt S, Detandt M (2012). The role of indoor pollution in the development and maintenance of chronic airway inflammation in children. B-ENT..

[16] United States Environmental Protection Agency. EPA Technology for Mold Identification and Enumeration. 2012. http://www.epa.gov. Accessed 2 June 2014.

[17] Jurjevic Z, Peterson SW, Horn BW (2012). *Aspergillus* section *Versicolores*: nine new species and multilocus DNA sequence based phylogeny. IMA Fungus.

[18] Jurjevic Z, Peterson SW, Solfrizzo M, Peraica M (2013). Sterigmatocystin production by nine newly described *Aspergillus* species in section *Versicolores* grown on two different media. Mycotoxin Res.

[19] Rank C, Nielsen KF, Varga J, Samson RA, Frisvad JC, Larsen TO (2011). Distribution of sterigmatocystin in filamentous fungi. Fungal Biol.

[20] Reed GH, Kent JO, Wittwer CT (2007). High-resolution DNA melting analysis for simple and efficient molecular diagnostics. Pharmacogenomics J.

[21] Nemcova E, Cernochova M, Ruzicka F, Malisova B, Freiberger T, Nemec P (2015). Rapid identification of medically important *Candida* isolates using high resolution melting analysis. PLoS One.

[22] Lengerova M, Racil Z, Hrncirova K, Kocmanova I, Volfova P, Ricna D (2014). Rapid detection and identification of *Mucormycetes* in bronchoalveolar lavage samples from immunocompromised patients with pulmonary infiltrates by use of high-resolution melt analysis. JCM..

[23] Somogyvari F, Horvath A, Serly J, Majoros H, Vagvolgyi C, Peto Z (2012). Detection of invasive fungal pathogens by real-time PCR and high-resolution melting analysis. In vivo.

[24] Alonso M, Escribano P, Guinea J, Recio S, Simon A, Peláez T (2012). Rapid detection and identification of *Aspergillus* from lower respiratory tract specimens by use of combined probe-high-resolution melting analysis. JCM.

[25] Biorad. Precision melt analysis ™ : Instruction manuel.2012 http://www.bio-rad.com/webroot/web/pdf/lsr/literature/Bulletin_10014811.pdf. Accessed Mar 2016.

[26] van den Berg M, Albang R, Albermann K, Badger J, Daran J-M, Driessen A (2008). Genome sequencing and analysis of the filamentous fungus *Penicillium chrysogenum*. Nat Biotechnol.

[27] Hyvarinen A, Vahteristo M, Melkin T, Jantunen M, Nevalainen A, Mougel C (2001). Temporal and spatial variation of fungal concentrations in indoor air. Aerosol Sci Technol.

[28] Benndorf D, Müller A, Bock K, Manuwald O, Herbarth O, von Bergen M (2008). Indentification of spore allergens from the indoor mould *Aspergillus versicolor*. Allergy.

[29] Reboux G, Bellanger AP, Roussel S, Grenouillet F, Sornin S, Piarroux R (2009). Indoor mold concentration in eastern France. Indoor Air.

[30] Jones R, Recer GM, Hwang SA, Lin S (2011). Association between indoor mold and asthma among children in buffalo. New York Indoor Air.

[31] Meheust D, Le CP, Reboux G, Millon L, Gangneux JP (2014). Indoor fungal contamination: health risks and measurement methods in hospitals, homes and workplaces. Crit Rev Microbiol.

[32] Vesper SJ, Wymer L, Kennedy S, Grimsley LF (2013). Decreased pulmonary function measured in children exposed to high environmental relative moldiness index homes. Open Respir Med J.

